# A multitaxon biodiversity dataset from hemiboreal forests of different stand ages and management histories

**DOI:** 10.1016/j.dib.2026.112862

**Published:** 2026-05-18

**Authors:** Piret Lõhmus, Asko Lõhmus, Ann Kraut, Liina Remm, Leif-August Kirs, Anneli Palo, Eliisa Pass, Ekaterina Rozantseva, Valentina Zolotarjova, Maarja Vaikre, Kadri Runnel

**Affiliations:** a Institute of Ecology and Earth Sciences, University of Tartu, J. Liivi 2, 50409 Tartu, Estonia; b Estonian Natural History Museum, Lai 29a, 10133 Tallinn, Estonia

**Keywords:** Beetles, Boreo-nemoral, Bryophytes, Lichens, Molluscs, Wood fungi, Snails, Vascular plants

## Abstract

**Data description:**

This dataset compiles standardized multitaxon biodiversity data from >300 forest stands in the European hemiboreal region. It includes species lists and species-level abundance data for six groups — beetles, bryophytes, lichens, polypore fungi, molluscs, and vascular plants — surveyed across a wide range of forest types, stand ages, and management histories. Species identifications are of high taxonomic resolution; for polypores and lichens, morphological determinations were often complemented by DNA barcoding or chemical analyses to ensure accuracy. A subset of stands has been surveyed repeatedly, enabling assessment of temporal changes in forest biodiversity.

**Study area:**

Estonia, hemiboreal forest zone. Field sampling was conducted between 2005 and 2025.

**Data coverage:**

The dataset covers 338 forest stands (including recent cutover stands) represented by 2-ha plots and includes records of >2200 taxa across six major groups. All six groups were recorded in 16% of the stands, and half of the stands include data for at least three groups, with the most complete coverage for polypores and lichens. The stands represent the major hemiboreal forest types along gradients of dominant tree species and soil fertility, ranging from low-productivity dry Scots pine forests to black alder–dominated swamps, with strong representation of productive Norway spruce–deciduous mixed forests. Stand ages range from recent post-clearcut stages to forests with dominant tree layers exceeding 200 years. Old stands without signs of management are distinctly represented and serve as natural baselines for old-growth biodiversity in the region. This is the most comprehensive and representative multitaxon forest biodiversity dataset currently available from the European hemiboreal zone.

**Methods:**

Within each species group, field sampling was performed by experts and followed consistent standardized protocols across study stands, yielding comparable species-level abundance data.

**Applications:**

The dataset enables analyses of within- and cross-taxon biodiversity patterns in relation to forest type, stand age, and management history. Combined with detailed forest structure data available for most study stands from a parallel published dataset, it enables linking biodiversity variables to habitat availability and above-ground tree carbon. Geopositioned plots facilitate integration with spatial layers and remote sensing products, enabling extrapolation of biodiversity–habitat (or aboveground tree carbon) relationships across larger forest landscape mosaics, also beyond the hemiboreal region. The temporal span of the dataset enables both repeated surveys of the same stands to assess biodiversity change over time and comparisons with stands of similar characteristics to infer broader temporal shifts in forest biodiversity. The strong representation of old-growth stands establishes a robust natural baseline for evaluating biodiversity responses to forest management and long-term landscape change.

Specifications Table

**Table:** 

Subject	Biology
Specific subject area	Biodiversity data covering beetles, land molluscs, vascular plants, bryophytes, lichens and polypores across 338 forests of different hemiboreal forest types and stand ages.
Type of data	Raw multitaxon biodiversity data (species lists with abundance estimates), geospatial data (map layer with stand polygons).
Data collection	Data were collected during multiple field campaigns between 2005 and 2025. Species lists and abundance data for sessile organisms (bryophytes, lichens, polypores, and vascular plants) were obtained using a standardized fixed-area, fixed-effort approach. Beetles were sampled using flight interception traps, complemented by rearing from downed wood in a subset of study stands. Land molluscs were surveyed using a combination of litter extraction and visual search.
Data source location	Country: Estonia (57.3-59.5 ° N; 21.5-28.1 ° E). Precise locations of study stands are provided on a GIS map layer with plot polygons.
Data accessibility	Repository name: Zenodo Data identification number: 10.5281/zenodo.19370022 Direct URL to data: https://doi.org/10.5281/zenodo.19370022
Related research article	Diverse research articles.

## 1 Value of the Data

• This is the most comprehensive multitaxon forest biodiversity dataset currently available from the European hemiboreal region, covering six ecologically distinct species groups that represent different dimensions of forest ecosystem functioning and habitat provisioning. Species identifications are of high taxonomic resolution; for example, approximately 10% of polypore identifications have been verified using DNA barcoding in addition to micromorphological identification.
• The dataset spans a broad range of forest types and management histories, including recently clearcut sites with and without retention trees, mature managed forests, old-growth stands, and drained forest ecosystems. Together with repeated surveys in a subset of stands, this enables an overview of hemiboreal forest biodiversity. Measurements beginning in the early 2000s provide historical reference points for long-term monitoring and retrospective analyses.
• A substantial proportion of the studied stands (80%) is represented in a parallel published dataset [1] that provides detailed stand structure information, including deadwood availability and live-stand characteristics. Together, these datasets allow robust analyses of relationships between biodiversity, forest structure, management history, stand age, and above-ground tree carbon across diverse hemiboreal forest types.
• A spatial layer with plot polygons enables direct linkage of each field-measured stand to airborne and satellite data from the corresponding inventory year. This supports spatial modelling, landscape-scale extrapolation, and calibration of remote-sensing-based biodiversity and habitat quality estimates.
• For lichens, bryophytes, and polypore fungi, stand-level abundance data are complemented by precise substrate-level occurrence records (e.g., host tree species, living or dead tree, dead wood type, decay stage). This allows analyses of species assemblages within specific microhabitats and facilitates flexible comparisons across regions where habitat availability differs.
• The sessile species groups (lichens, bryophytes, polypores, and vascular plants) were sampled using a fixed-area, fixed-effort protocol [2] that also records the time of first detection for each species. This enables effort-standardized biodiversity comparisons and the construction of observer-specific species accumulation curves, allowing potential observer bias to be assessed and accounted for.

## 2 Background

Under natural conditions, forests would dominate large areas of boreal and temperate regions [3]. Extensive human land use has greatly reduced forest cover and reshaped most remaining forests through management, leading to fragmentation, simplified stand structure, and younger age distributions compared to natural baselines [4]. These structural changes are known to affect biodiversity, yet comprehensive assessment remains challenging because many forest-dwelling organisms are difficult to detect and identify.

Over the past two decades, we have systematically surveyed multiple understudied groups of forest taxa across different hemiboreal forest conditions. The dataset presented here consolidates these long-term survey efforts into a unified and standardized resource.

Subsets of these data have previously been used to assess habitat value and drivers of biodiversity patterns across managed and old-growth hemiboreal forests , including the effects of seminatural forest management history [[5], [6], [7], [8], [9], [10], [11], [12]], long-term habitat protection [13,14], and the potential threats posed by intensified forest management practices [15]. The data have also enabled larger regional syntheses of biodiversity patterns and management effects on the focal species groups [9,16,17] and have served as a basis for Red List assessments and biodiversity conservation planning [[16], [17], [18], [19]]. In addition, the data have been used in methodological studies assessing species-detection challenges in forest cryptogams and the resulting biases [20,21], and comparing visual species surveys with eDNA-based methods [22,23].

## 3 Data Description

**Fig. 1:**
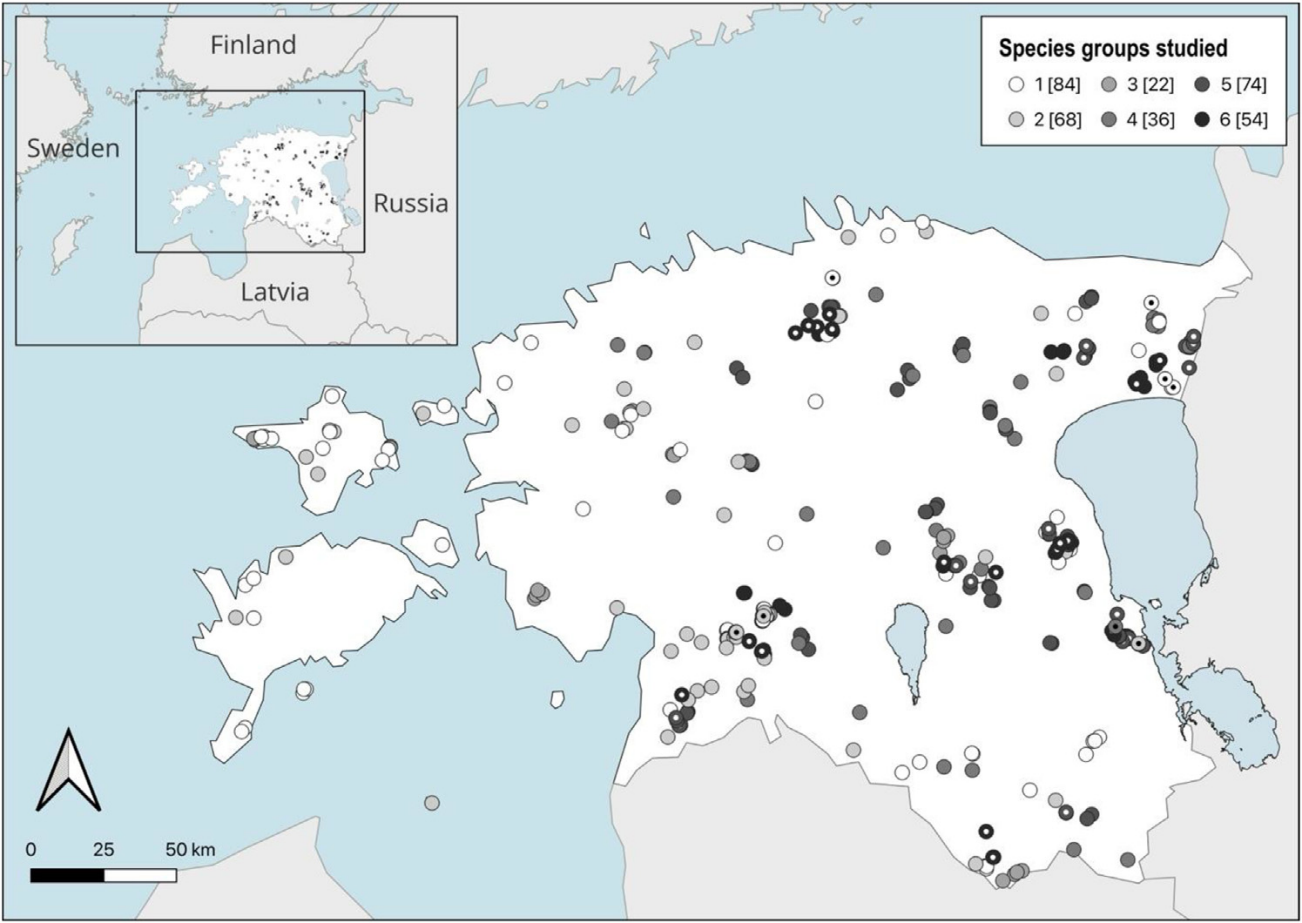
Locations of the studied forest stands in Estonia. Symbol shading indicates the number of species groups surveyed in each stand; numbers in square brackets show the number of stands in each group. Points with central dots denote stands where biodiversity surveys have been repeated one or more times (see Table 1.1 in the published dataset).

This article describes the dataset available in the Zenodo repository [27], which contains multitaxon forest biodiversity data from Estonian hemiboreal forests across different forest types, successional stages, and forest management backgrounds (Table 1). The data were collected in 338 forest stands between 2005 and 2025 (Fig. 1). The repository includes one GIS map layer, a README file in (.txt format), a table explaining taxonomy (in .xlsx format) and two Excel workbooks, each containing multiple worksheets. An overview of the contents of these files is provided in Table 2.

**Table 1:** Overview of the studied species groups, forest types, successional stages and forest management conditions across the different subsets of the data. The abbreviations for successional stages are: E, early successional; Mi, middle aged; Ma, mature; O, old. The subset numbers and names correspond to those used in [1]. The standardized survey methods allow comparisons across subsets.

Sub-set	Subset name	Species groups	Site types	Successional stage	Management background	No. of stands	Study year	References
1	LIST	Beetles, bryophytes, lichens, land molluscs, polypores, vascular plants	Alvar*, Dry boreal, Meso-eutrophic, Eutrophic, Swamp, Drained peatland	E, Mi, Ma, O	Clearcut, green tree retention, commercial thinning, slash harvesting. Control: old natural stands.	160	2005–2020#	[5,6,[9], [10], [11], [12],^15^]
2	DREX	Polypores, vascular plants	Drained peatland	Mi, Ma	2013: drainage for forestry 2018: ditch closure, commercial thinning, gap harvesting, understory removal for wetland forest restoration	48	2013, 2014, 2018##	[24,25]
3	CICNIG	Bryophytes, lichens, polypores, vascular plants	Meso-eutrophic, Eutrophic, Drained peatland	Ma, O	Protection for an iconic bird species.	20	2014, 2016	[13]
4	RMK forest fragments	Lichens, polypores	Dry boreal, Meso-eutrophic, Eutrophic, Paludifying, Swamp, Drained peatland	Ma, O	Edge effects: thinning and clearcuts in adjacent stands. Control: old natural stands.	26	2019–2020	[23]
11	BURN	Lichens	Dry boreal, Paludifying	E, Mi, Ma	After fire: natural regeneration (recent and old burnings), salvage logging.	9	2010- 2011, 2015	[8]
12	LaSa	Beetles, land molluscs, lichens, vascular plants	Meso-eutrophic, Eutrophic	E, Mi, Ma, O	Planting vs. natural regeneration. Control: old natural stands.	40	2020–2021	[26]
13*	VARIA	Bryophytes, lichens	Dry boreal, Meso-eutrophic, Eutrophic, Eutrophic_paludifying, Swamp, Drained peatland	Mi, Ma, O	Protection for an iconic moss.	35	2019, 2022, 2025	[14]
**TOTAL**						**338**		

# 58 stands surveyed twice (interval 6–15 years, depending on species group); 8 stands surveyed 3–8 times for polypores.

## a subset of 16 stands studied two times: before and after the management treatments.

⁎ not represented with stand stucture data in a parallel dataset [1].

**Table 2:** Content of the text and data files of the dataset (DOI: 10.5281/zenodo.19370022).

Table	Title	Content
0.0	README.txt	Overview of the dataset and repository structure. Describes all tables and explains linking keys used across tables. Also describes the GIS data, including geometry type, coordinate reference system and projection, and the identifiers used to link spatial features to tabular data.
0.1	taxonomy.xlsx	Lists taxa with problematic or unresolved taxonomy, and explains how such cases were treated.
1_subsets_stands (an Excel file with three worksheets)		
1.0	metadata_subsets_ stands	Describes all variables, defines terms and abbreviations, specifies measurement units in tables 1.1 and 1.2
1.1	subsets	Overview of the original studies contributing to the dataset, including subset ID, study period, and the successional stages and forest management conditions covered. This table expands the information summarised in Table 1 in the manuscript.
1.2	stands	Data for each study stand, including stand ID (linked to the GIS map layer), year of stand origin, stand successional stage at sampling, forest site type, centroid coordinates, and survey year of particular species group. Species-level abundance data for all recorded taxa across studied species groups: abundance classes for sessile organisms; counts of individuals per species for beetles and molluscs. For sessile organisms, the data includes time of first detection during fixed-time surveys. For stands inventoried on multiple occasions, all data is reported separately for each inventory year.
2_details (an Excel file with six worksheets)		
2.0	metadata_detailed	Describes all variables, defines terms and abbreviations, specifies measurement units in tables 2.1–2.5.
2.1	bryophyte_detailed	Records of bryophyte species in each studied stand by different growing substrates (forest ground, live trees of different tree species, snags or downed dead wood of different decay stages).
2.2	lichen_detailed	Records of lichen species in each studied stand by different growing substrates (forest ground, live trees of different tree species, snags or downed dead wood of different decay stages).
2.3	polypore_detailed	Individual polypore records in each studied stand, with substrate identity (forest ground, live trees of different species, snags or downed dead wood of different decay stages) and diameter at the point of fruit-body formation. For abundant species, only the first ten substrates are given.
2.4	beetle_detailed	Beetle taxon records from individual flight interception traps in each studied stand, and taxa obtained by rearing from downed wood in a subset of stands.
2.5	mollusc_detailed	Mollusc records split into juvenile live, juvenile empty, adult live, and adult empty individuals.

The dataset is organised into two data levels: study-stand level and individual-object level. Stand-level data provide species lists and species-level abundance estimates for all studied taxa and, for sessile organisms, their discovery time (first detection, 0.5 h resolution) during fixed-time surveys. The latter enables comparisons with studies using different survey effort and construction of recorder-specific species accumulation curves (for assessing and accounting for potential observer bias). Nomenclature (Latin names) follows the GBIF Backbone Taxonomy.

In addition, more detailed tables are provided for polypores, lichens, bryophytes, beetles and land molluscs. For polypores, lichens, and bryophytes, these tables contain substrate-level records from each study stand. These enable subsetting the data by specific substrates (e.g. tree species, deadwood type such as snags or downed deadwood, and dead wood decay stage) and estimating the relative biodiversity contribution of these substrates in forest landscapes. For beetles, a more detailed table contains records from individual flight interception traps, enabling comparisons with studies that use different numbers of traps, and additionally includes species obtained by rearing from downed dead wood in a subset of study stands. For land molluscs, the detailed table provides separate counts for four categories: juvenile live, juvenile empty, adult live, and adult empty individuals.

## 4 Experimental Design, Materials and Methods

### 4.1 Study region and forest types

The study was conducted in Estonia, a lowland country located in the European hemiboreal forest zone along the Baltic Sea. Detailed descriptions of the study region, forest site-type classification, successional stages, and forest management context are provided in a parallel dataset describing forest structure in a largely overlapping set of study stands [1], with 54 additional stands included here.

Estonian forest ecosystem composition and dynamics are primarily organized along soil fertility and moisture gradients, as typical in the hemiboreal region. The dataset includes stands representing the main forest site-type groups: (1) dry boreal Scots pine (*Pinus sylvestris*) forests on nutrient-poor mineral soils; (2) meso‑eutrophic mixed forests; (3) eutrophic boreo-nemoral mixed or deciduous dominated forests on fertile mineral soils; (4) eutrophic paludifying forests on wet mineral or shallow peat soils; (5) swamp forests dominated by black alder (*Alnus glutinosa*) or downy birch (*Betula pubescens*); (6) drained peatland forests that have developed after draining forested or open mires for forestry; and (7) Norway spruce (*Picea abies*) dominated alvar forests on calcareous soils. These forest ecosystems span a wide range of soil productivity conditions and together represent much of the ecological variation that can be found in European hemiboreal forests. Importantly for biodiversity patterns, 85% of the forest stands in this dataset were situated in continuous forest land (i.e., areas that have remained forested at least over the past century); the remaining stands were, a hundred years ago, either mires with sparse tree and shrub cover or seminatural grasslands.

### 4.2 Stand successional stages

The sampled stands cover all main successional stages of hemiboreal forests, ranging from early post-clearcut stages to old stands exceeding 200 years of age. Stand development stages follow the commonly used Estonian forestry classification, in which early-successional stands occur during the first decades after clearcutting (nowadays with some tree-retention), followed by middle-aged and mature stages, while old stands represent late-successional forests. A subset of old stands without visible signs of management is treated as natural reference stands representing old-growth forest conditions. Detailed definitions of these stages and the underlying productivity-based age thresholds are provided in [1].

### 4.3 Forest management background

Most of the studied managed stands originate from the even-aged production-forestry system nowadays dominant in Estonia; in this system, clearcutting is followed by regeneration either through planting or natural regeneration and subsequent stand tending and thinning. Early-successional stands in the dataset therefore mostly represent post-clearcut forests, often with green-tree retention. Middle-aged and mature stands include forests with diverse management histories, including planted and naturally regenerated stands, forests subjected to commercial thinning, stands influenced by adjacent clearcuts, and in some cases restoration treatments such as ditch closure in drained peatlands. Burned stands resulted from accidental human-initiated fires.

### 4.4 Fieldwork

The primary sampling unit was the forest stand, as delineated in the Estonian Forestry Registry. It refers to a forest landscape unit that has a more or less uniform site type, stand age, and overstorey tree species; it is also the management unit in even-aged silviculture. In all studied stands, biodiversity surveys were conducted within a 2-ha plot in the stand delineated on the map prior to field sampling; a standard plot size was important to represent similar extent of spatial variability. Rectangular plots were preferred, although landscape features occasionally required more complex shapes.

#### 4.4.1 Sessile organisms (vascular plants, bryophytes, lichens, polypores)

The four sessile species groups were surveyed following a standardised fixed-area-fixed-effort protocol [2]. ‘Lichens’ comprised both macro- and microlichens, lichenicolous calicioids, and certain saprotrophic microfungi taxa traditionally studied by lichenologists. Bryophytes comprised both mosses and hepatics. Vascular plants comprised trees, shrubs, and herbaceous species. In each 2-ha plot, the full assemblage of each species group was inventoried during a fourhour survey conducted in the appropriate season (vascular plants in June–August; bryophytes and lichens during the snow-free period; polypores in September–October). All types of suitable substrates up to 2 m height from the forest floor were checked, with the primary aim of finding as many species as possible. According to field estimates, this approach usually reveals > 70% of all species present in the 2-ha plot, ranging from ca. 50% in lichens and polypores in the most diverse forests to > 90% of vascular plants in species poor stands [2].

For each detected species, we categorized its abundance on an approximately logarithmic scale. For bryophytes, lichens, and polypores, a five-point scale was used (1, one record; 2, 2–5 records; 3, 6–15 records; 4, 16–100 records; 5, > 100 records). For vascular plants, a ten-point scale was used, ranging from one shoot (score 1), 2–3 scattered shoots or a clone (score 2) to local dominance (score 8) or total dominance (score 9 for > 50% total cover; score 10 for > 90% cover); approximate percent cover estimates for those classes are presented by [24]. Note that while species lists for vascular plants are comparable across the dataset, abundance data for trees and shrubs are not available for one of the subsets.

Where necessary, specimens that could not be identified in the field were collected and examined in the laboratory. Identification methods included microscopy, thallus color spot tests and thin-layer chromatography to detect lichen compounds or, in the case of polypores, sequencing of the rDNA ITS region for comparison with reference sequences.

#### 4.4.2 Land molluscs

The 2-ha plots were sampled in early autumn within a short time period, and adjascent stands (blocks containing clearcut and mature managed stands, and old-growth controls) were usually sampled on the same day. Three liters of litter and topsoil, passed through a sieve with a 1-cm mesh, were collected from each plot. The material was collected as six 0.5-l subsamples, each obtained by haphazard sampling of different microhabitats while walking slowly throughout the 2-ha plot, and combining collection with a simultaneous visual search [28]. In the laboratory, snails were sorted and identified based on morphological characteristics [11]. Juveniles and adults were recorded separately, and both live individuals and empty shells were counted (data provided both as total counts per species and as separate counts for juvenile live, juvenile empty, adult live, and adult empty individuals).

#### 4.4.3 Beetles

In each 2-ha plot, beetles were caught with two flight-intercept traps consisting of two transparent plastic sheets (25 × 40 cm) attached vertically on top of a plastic funnel (diameter 25 cm) filled with NaCl-solution and detergent as surfactant. The traps were attached on live trees or snags at breast height on sun-exposed side, 40–100 m from each other, and at least 20 m from stand edge. Traps were emptied monthly, in most cases from May until the end of September. Collected insects were stored in 70% ethanol [5,15].

The nomenclature for all studied species groups follows the GBIF Backbone Taxonomy (as of March 2026), and the data tables also provide the numerical GBIF Taxon Key, a GBIF identifier used to represent species or other taxonomic levels. Collected specimens have been deposited in the collections of the University of Tartu Natural History Museum (TUF) and the Estonian University of Life Sciences (TAA). For polypores, rDNA ITS sequences are stored in the UNITE database; voucher specimen numbers and the availability of DNA data are indicated in table 2.3 of the dataset.

## 5 Limitations

A general limitation of biodiversity data is that not all species present in a stand are detected during surveys, and this applies also to the present dataset. This is particularly relevant for rare and inconspicuous species, which are more likely to remain undetected. Nevertheless, at least for sessile species groups, the standardized and relatively intensive sampling protocols used here provide substantially more complete species inventories than most other biodiversity surveys at similar spatial scales [2]. This allows the use of species absence data in analyses, but potential detection errors should be considered, especially for rare species.

A second limitation is that forest structure data from a parallel dataset [1] are not available for 55 stands (16% of the stands presented here), which restricts direct linkage of biodiversity patterns to detailed structural variables for these sites. However, basic stand descriptors – including forest site type, dominant tree species, stand age, and management status — are available and provided for these stands, as well as the map polygons. These variables allow comparisons with other stands of similar characteristics and enable integration with external spatial data sources such as forest registry information or remote sensing products. In addition, the substrate-level records included for several species groups allow habitat availability to be summarized at the stand level even where detailed structural measurements are lacking.

## Ethics Statement

The authors have read and follow the ethical requirements for publication in Data in Brief and confirm that the current work does not involve human subjects, animal experiments, or any data collected from social media platforms.

## CRediT Author Statement

**Piret Lõhmus:** Conceptualization, Investigation, Data Curation, Writing - Review & Editing; Funding aquisition; **Asko Lõhmus:** Conceptualization, Data Curation, Methodology, Investigation, Writing – Review & Editing; Funding aquisition; **Ann Kraut:** Investigation, Data Curation, Writing - Review & Editing; **Liina Remm:** Investigation, Data Curation, Writing - Review & Editing; **Leif-August Kirs:** Investigation, Writing - Review & Editing; **Anneli Palo:** Investigation, Writing - Review & Editing; **Eliisa Pass:** Investigation, Writing - Review & Editing; **Ekaterina Rozantseva:** Investigation, Writing - Review & Editing; **Valentina Zolotarjova:** Investigation, Writing - Review & Editing; **Maarja Vaikre:** Investigation, Writing - Review & Editing; **Kadri Runnel:** Conceptualization, Investigation, Data Curation, Writing - Original draft, Project administration, Funding aquisition.

## Funding

This work was supported by 10.13039/501100005189Estonian Research Council [ETF6457, ETF7402, SF0180012s09, ETF9051, IUT34-7 and PRG1121 to AL; ETF7987 to PL; PSG825 and TK232 to KR]; Estonian State Forest Management Centre [LLOOM13051 to AL; LLOOM08244 to PL, and LLTOM18470 to KR]; Estonian Environmental Investment Centre [projects 11594 and 16288 to AL; 15431 to PL and 11061 to KR]; Estonian Environment Agency (LLOOM15080 to PL); Estonian Environmental Board (LLTOM16048, LLTOM21521 and LLOOM10074 to PL), and European Union through the European Regional Development Fund (the Centre of Excellence FIBIR).

## Data Availability

(Zenodo).Data from: A multitaxon biodiversity dataset from hemiboreal forests of different stand ages and management histories (Original data)
